# Minor Trauma Causing Stroke in a Young Athlete

**DOI:** 10.1155/2015/182875

**Published:** 2015-03-26

**Authors:** Vineet Gupta, Naveen Dhawan, Jaya Bahl

**Affiliations:** ^1^Department of Medicine, University of California San Diego (UCSD), San Diego, CA 92103, USA; ^2^Nova Southeastern University, 3301 College Avenue, Fort Lauderdale, FL 33314, USA; ^3^Florida International University (FIU), 11200 SW 8th Street, Miami, FL 33174, USA

## Abstract

A 17-year-old Caucasian male presented with sudden dizziness, ataxia, vertigo, and clumsiness lasting for a couple of hours. He had a subtle trauma during a wrestling match 2 days prior to the presentation. A CT Angiogram (CTA) and MRI showed left vertebral artery dissection (VAD). The patient was treated with anticoagulation with heparin drip in the ICU. The patient was discharged home on the third day on Lovenox-warfarin bridging. This case underscores the importance of considering VAD as a differential diagnosis in patients with sports-related symptoms especially in activities entailing hyperextension or hyperrotation of neck. Due to a varied latent period, often minor underlying trauma, and subtle presentation, a low index of suspicion is warranted in timely diagnosis and treatment of VAD. Considering recent evidence in treatment modality, either antiplatelet therapy or anticoagulation may be used for treatment of VAD.

## 1. Background

Vertebral artery dissection (VAD) is an underrecognized cause of stroke in patients younger than 45 years [1]. VAD is marked by several causes. It can be due to hyperextension of the neck by a variety of sports-related activities or cervical manipulation [2]. VAD is much less common than carotid artery dissection, but it can be a potentially life-threatening episode. The mortality rate of VAD in the acute phase is about 10% [1]. We present an interesting case of young male with subtle clinical manifestations who was later diagnosed with VAD.

## 2. Case Presentation

A 17-year-old Caucasian male backyard wrestler with no significant past medical history presented with acute onset of transient dizziness, ataxia, vertigo, and clumsiness lasting for a couple of hours while watching a baseball game. The patient complained of the onset of symptoms while turning his head to left. The patient was asymptomatic on presentation; the physical examination was unremarkable for any focal neurological deficits or constitutional symptoms.

During a thorough history-taking, it was revealed that the patient hyperextended his neck during a mock wrestling match 2 days prior to presentation. The patient did not develop any headache, loss of consciousness, or focal neurologic deficits at that time. The patient remained asymptomatic during the intervening days.

## 3. Investigations

**Figure 1:**
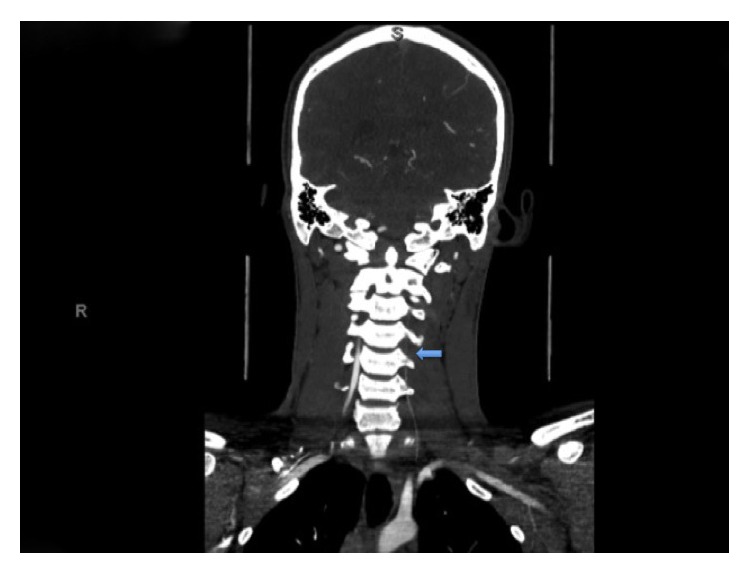
CT Angiogram shows attenuated filling of left vertebral artery (extracranial) almost in its entire length secondary to dissection (marked by solid arrow).

**Figure 2:**
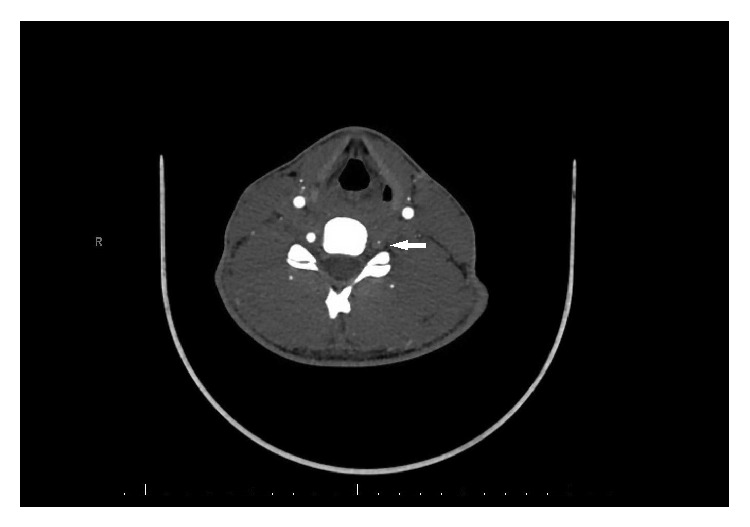
CT Angiogram showing the filling defect in left vertebral artery (marked by white solid arrow).

**Figure 3:**
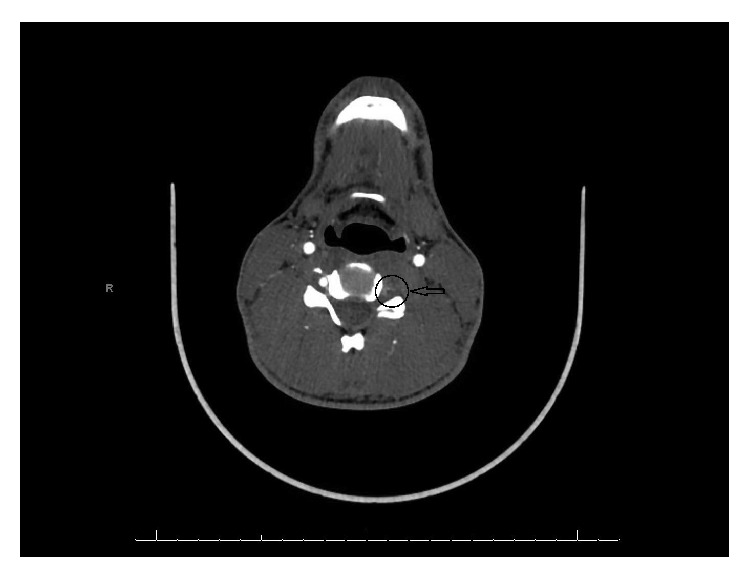
CT Angiogram showing hyperattenuating crescent shaped mural thickening of left vertebral artery (marked by black circle and arrow).

A CT scan head was unremarkable for any intracranial bleed. CT Angiogram (CTA) and MRI were notable for the dissection of almost the entire length of the left vertebral artery (extracranially) not involving the basilar artery (Figure 1). Dissection was further characterized by filling defect and mural thickening (Figures 2 and 3).

## 4. Differential Diagnosis

The differential diagnosis for the presenting symptoms of ataxia, vertigo, and dizziness includes stroke (hemorrhagic and ischemic), cervical spine fracture, cervical strain, subarachnoid hemorrhage, and atherothrombotic disease of the vertebrobasilar region and tension headache [1]. Migraine headaches must also be excluded as a cause [3]. VAD is associated with collagen disorders such as Ehlers-Danlos syndrome, Marfan's syndrome, and osteogenesis imperfecta which render the vertebral arteries more prone to dissection [4]. Additionally, hyperhomocysteinemia and MTHFR mutations may also lead to VAD [5].

## 5. Treatment

Given the absence of intracranial bleed on CT scan brain and primarily extracranial involvement of vertebral artery, the patient was started on a heparin drip for anticoagulation and admitted to the intensive care unit (ICU) for closer observation. The patient had unremarkable ICU course for any neurological changes. The patient was discharged home on the third day on Lovenox-warfarin bridging with follow-up CT Angiogram in 3 months.

## 6. Outcome and Follow-Up

When last contacted, the patient had been neurologically stable. He has not engaged in any wrestling activities and has not experienced any trauma.

## 7. Discussion

The underlying lesion in VAD is an expanding intramural hematoma, secondary to hemorrhage of the vasa vasorum within the media of vertebral artery, arising either spontaneously or after minor trauma [1]. Intimal disruption and decreased flow states predispose to a thrombogenic milieu that can lead to emboli formation and propagation.

It is estimated that VAD occurs at an incidence of about 1–1.5 per 100,000 [6]. Females are generally three times as likely to experience VAD as compared to males. The disease is usually seen in young patients (less than 45 years of age). The mortality rate in the acute phase is about 10% due to extensive intracranial dissection, brainstem infarction, or subarachnoid hemorrhage.

Causes of VAD include activities that entail hyperrotation or hyperextension of the neck, ranging from trivial activities to sports [7]. Trivial trauma may include spinal manipulation, nose blowing, yoga, and ceiling painting. Sports-related injuries that have been previously described include golf, judo, Kung Fu, and swimming [8–11]. Hyperextension of neck was noted to be the cause in our patient.

Unique anatomical attributes of vertebral arteries render them more prone to dissection than the carotid arteries. The vertebral arteries are particularly vulnerable to spinal compression, particularly at the level of the C1 and C2 vertebrae, where the rotation of the head takes place [12]. In contrast to the carotid artery, which is positioned in a less taut manner within the neck's soft tissue, the vertebral arteries traverse around the C1 vertebrae, making them more prone to injury caused by hyperextension or hyperrotation of the neck [7, 13].

Typical presentation of VAD includes severe occipital headache and posterior nuchal pain following a recent, relatively minor, head or neck injury involving some degree of cervical distortion [5]. Patients most commonly report neurological symptoms attributable to lateral medullary dysfunction as noted in our patient. The occipital and nuchal pain associated with VAD mimics musculoskeletal pain and often is attributed to the mechanical strain that precipitated the dissection. Thus, the symptoms are often mistaken for musculoskeletal prior to the development of neurological symptoms [5]. Focal neurological signs attributable to brainstem and cerebellum ischemia (typically signs of lateral medullary dysfunction) eventually develop in up to 85% of the patients. The latent period between trauma and neurological symptoms may be variable, ranging from days to weeks and even years [1].

CT Angiogram or MRA/MRI is the investigation of choice. MRI shows vertebral arteries that are enlarged with a rim of hyperintense signal around a hypointense signal (lumen) [5].

Treatment for VAD lacks consensus with the option of using either anticoagulation or antiplatelet therapy for stroke prevention [14]. Rationale for anticoagulation is to prevent thrombogenic or embolic occlusion in the intimal flap that results from the separation of the intima from the vascular wall [11]. It is typically instituted for 3–6 months after ruling out subarachnoid hemorrhage or intracranial VAD [11]. Antiplatelet therapy offers a less risky alternative for bleeding complications. Adding to the pool of evidence, the maiden randomized trial CADISS found no difference in efficacy of antiplatelet and anticoagulant therapy in preventing stroke and death in patients with symptomatic carotid and vertebral artery dissection [14, 15]. Currently, the VAD therapy is tailored on a case-by-case basis and is often influenced by the experience and preference of the treating physician.

In a young individual less than 45 years of age presenting with stroke-related symptoms, a diagnosis of VAD can be easily mistaken for other more common causes of stroke. This case underscores the importance of considering VAD on the list of differentials when evaluating symptoms related to sports activities. In particular, a thorough history is essential and one must consider hyperextension or hyperrotation of the neck as part of the patient's activity that may have caused VAD.

## 8. Learning Points

- VAD is an underrecognized cause of stroke especially in younger age groups (less than 45 years of age).
- VAD should be considered in symptomatic cases of hyperextension or hyperrotation of the neck during sports-related activities.
- Due to a varied latent period, minor underlying trauma, and subtle presentation, a thorough history and strong index of suspicion are necessary for its timely diagnosis and treatment.
- Antiplatelet therapy and anticoagulation are equally efficacious for the prevention of stroke in VAD.

## References

[1] Lang E. S. (2014). *Vertebral Artery Dissection*.

[2] Albuquerque F. C., Hu Y. C., Dashti S. R. (2011). Craniocervical arterial dissections as sequelae of chiropractic manipulation: patterns of injury and management. *Journal of Neurosurgery*.

[3] Pezzini A., Granella F., Grassi M. (2005). History of migraine and the risk of spontaneous cervical artery dissection. *Cephalalgia*.

[4] Grond-Ginsbach C., Debette S. (2009). The association of connective tissue disorders with cervical artery dissections. *Current Molecular Medicine*.

[5] Debette S., Leys D. (2009). Cervical-artery dissections: predisposing factors, diagnosis, and outcome. *The Lancet Neurology*.

[6] Redekop G. J. (2008). Extracranial carotid and vertebral artery dissection: a review. *Canadian Journal of Neurological Sciences*.

[7] Dziewas R., Konrad C., Dräger B. (2003). Cervical artery dissection—clinical features, risk factors, therapy and outcome in 126 patients. *Journal of Neurology*.

[8] Maroon J. C., Gardner P., Abla A. A., El-Kadi H., Bost J. (2007). ‘Golfer's stroke’: golf-induced stroke from vertebral artery dissection. *Surgical Neurology*.

[9] Lannuzel A., Moulin T., Amsallem D., Galmiche J., Rumbach L. (1994). Vertebral-artery dissection following a judo session: a case report. *Neuropediatrics*.

[10] Pacei F., Valvassori L., Bet L. (2014). Vertebral artery dissection during Kung-Fu training. *Neurological Sciences*.

[11] Ning M., Gonzalez R. G. (2013). Case 34-2013—a 69-year-old man with dizziness and vomiting. *The New England Journal of Medicine*.

[12] Amin F. M., Larsen V. A., Tfelt-Hansen P. (2013). Vertebral artery dissection associated with generalized convulsive seizures: a case report. *Case Reports in Neurology*.

[13] Menon R. K., Norris J. W. (2008). Cervical arterial dissection. Current concepts. *Annals of the New York Academy of Sciences*.

[14] Kasner S. E. (2015). CADISS: a feasibility trial that answered its question. *The Lancet Neurology*.

[15] The CADISS trial investigators (2015). Antiplatelet treatment compared with anticoagulation treatment for cervical artery dissection (CADISS): a randomised trial. *The Lancet Neurology*.

